# Microglial knockdown does not affect acute withdrawal but delays analgesic tolerance from oxycodone in male and female C57BL/6J mice

**DOI:** 10.3389/adar.2022.10848

**Published:** 2022-12-16

**Authors:** Omar El Jordi, Kathryn D. Fischer, Timothy B. Meyer, Brady K. Atwood, Adrian L. Oblak, Raymond W. Pan, David L. McKinzie

**Affiliations:** ^1^ Department of Pharmacology and Toxicology, Indiana University, Indianapolis, IN, United States; ^2^ Department of Radiology and Imaging Sciences, Indiana University, Indianapolis, IN, United States

**Keywords:** behavior, withdrawal, microglia, opioids, oxycodone, dependence, PLX3397, neuroinflammation

## Abstract

Opioid Use Disorder (OUD) affects approximately 8%–12% of the population. In dependent individuals, abrupt cessation of opioid taking results in adverse withdrawal symptoms that reinforce drug taking behavior. Considerable unmet clinical need exists for new pharmacotherapies to treat opioid withdrawal as well as improve long-term abstinence. The neuroimmune system has received much scientific attention in recent years as a potential therapeutic target to combat various neurodegenerative and psychiatric disorders including addiction. However, the specific contribution of microglia has not been investigated in oxycodone dependence. Chronic daily treatment with the CSF1R inhibitor Pexidartinib (PLX3397) was administered to knockdown microglia expression and evaluate consequences on analgesia and on naloxone induced withdrawal from oxycodone. In vivo results indicated that an approximately 40% reduction in brain IBA1 staining was achieved in the PLX treatment group, which was associated with a delay in the development of analgesic tolerance to oxycodone and maintained antinociceptive efficacy. Acute withdrawal behavioral symptoms, brain astrocyte expression, and levels of many neuroinflammatory markers were not affected by PLX treatment. KC/GRO (also known as CXCL1) was significantly enhanced in the somatosensory cortex in oxycodone‐treated mice receiving PLX. Microglial knock-down did not affect the expression of naloxoneinduced opioid withdrawal but affected antinociceptive responsivity. The consequences of increased KC/GRO expression within the somatosensory cortex due to microglial reduction during opioid dependence are unclear but may be important for neural pathways mediating opioid‐induced analgesia.

## Introduction

Opioids are the gold standard for pain management of serious pain conditions. Their superior efficacy at attenuating pain results in their frequent and typically long-term prescription for chronic pain states. Unfortunately, 8%–12% of people prescribed long-term opioid use become dependent and diagnosed with opioid use disorder (OUD) (1). In dependent individuals, withdrawal following cessation of opioid use is associated with a spectrum of adverse gastrointestinal, somatic, and autonomic symptoms and can be life-threatening in severe cases (2). Acute opioid withdrawal can be treated with the use of other opioids and benzodiazepines but carries additional safety concerns. Currently, the only non-opioid agent approved by the FDA to treat acute opioid withdrawal is the adrenergic α2 agonist lofexidine. While effective in ameliorating many autonomic-related symptoms, lofexidine is approved only for short-term use. For long-term OUD treatment, replacement (e.g., methadone and buprenorphine) and opioid blocking (e.g., naltrexone) therapies are used to maintain abstinence; however, several limitations of these treatments result in significant unmet clinical need (3). New therapeutic approaches are desperately needed that address the underlying biology mediating opioid dependence.

The intertwining of pain conditions and drug dependence/withdrawal is not surprising considering the overlap between the pathways. The analgesic and addictive attributes of common opioids are largely driven by the mu opioid receptor (MOR) subtype. MORs are G-coupled receptors that regulate expression of proteins central to neuronal activity such ion co-transporters (K+/Cl-), glutamate transporter (GLT-1), glutamate receptors (NMDA) among other mediators and targets (3–7). However, evidence suggests that glial cells (astrocytes and microglia) also express MOR where they indirectly regulate neuronal excitation (e.g., glutamatergic/GABA-ergic tone) as well as glial reactivity (pro-inflammatory cytokine and chemokine production). This is supported by multiple studies implicating glia in mediating pain signaling, opioid tolerance, and dependence (8–13). For example, chronic morphine treatment increases astrocytic reactivity markers (GFAP), microglial reactivity markers (IBA1), and cytokines/chemokines (IL-1B, fractalkine, BDNF, IL-10) in mice and rats (4, 12). Antagonizing inflammatory cytokines such as IL-1 or using non-selective glial inhibitors (ibudilast; minocycline; fluorocitrate) have been effective at decreasing analgesic tolerance and/or withdrawal symptoms (3, 4, 12, 14). Focused approaches on microglial MOR specific knockout highlighted the significance of these cells to morphine analgesic tolerance and withdrawal (12). This establishes the role of glia in opioid analgesic response however is challenged by differential efficacy profiles and opioid receptor subtype affinities of opioids (15, 16). This lays the foundation to tackle the unanswered question as which glial cell mediates withdrawal and analgesia in a clinically relevant opioid, oxycodone. We chose the somatosensory cortex and hippocampus due to their relevance in pain and reward pathways and high microglia expression (17, 18).

In this study we sought to understand the contribution of microglia on the development of opioid dependence and acute withdrawal in mice by selectively down-regulating microglia with PLX3397 (PLX) using an established 22-day treatment regimen (19, 20). PLX3397 was an ideal choice because it prevents the phosphorylation of the colony stimulating factor 1 tyrosine receptor (CSF1R) inhibiting the downstream expression of genes in macrophages/microglia critical for their viability (21). Opioid dependence was induced using a modified escalating dose regimen of Oxycodone (Oxy) over the final 9 days of PLX treatment. Various physiological and behavioral measures were assessed during opioid dependence development that culminated in evaluation of naloxone-induced withdrawal severity (22). Somatosensory cortex and hippocampus were dissected for analysis of microglia and astrocyte expression levels and levels of key inflammatory proteins were determined.

## Materials and methods

### Animal use

Male and female C57BL6/J mice were purchased from Jackson Labs. Male and female mice were 14–16 weeks old and weighed on average 27 and 21 g, respectively. Mice were maintained in a temperature controlled (21 ± 2°C) and 12-h light/dark cycle (lights on at 0700). Mice were provided *ad libitum* water (reverse osmosis city water) and standard chow (Teklad Cat# 2018SX) throughout the treatment. All animal work, paradigms, treatments, and diets were approved by the Institutional Animal Care and Use Committee (IACUC) at the Indiana University School of Medicine and align with the guidelines established by NIH for animal research.

### Drugs and chemicals

PLX3397 was purchased from MedChemExpress (Cat# HY-16749) Peanut butter balls were made using creamy Jif brand peanut butter spread mixed with finely chopped oatmeal (66%:33%, respectively) and saline (0.2 ml for every 0.750 g of mixture). Each mouse was given 0.5 g peanut butter ball once a day which delivered 40 mg/kg/day of PLX (23). Mice consumed the daily peanut butter ball within 5–10 min of delivery to the home cage. Oxycodone HCl was obtained from the NIDA drug supply program. 2,2,2-tribromoethanol was purchased from VWR (Cat# T1686). Prolong Gold Antifade with DAPI from Thermofisher (Cat# P36931). Formula 83 (Cat# NC0406699) was purchased from Biotech.

### Oxycodone dependence and naloxone precipitated withdrawal paradigm

**FIGURE 1:**
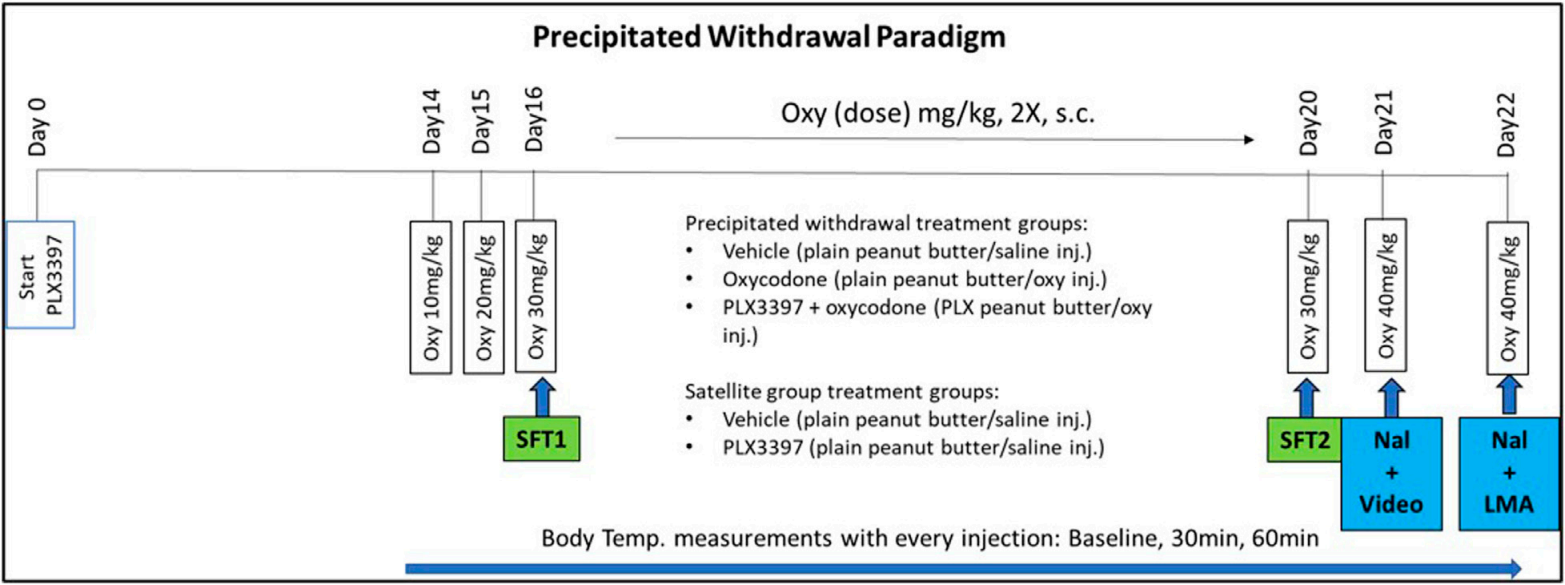
PLX3397 and oxycodone treatment paradigm timeline. Mice were treated with PLX3397 from Day0 to Day22. On Day14, they began an escalating oxycodone treatment until the day of euthanization (Day22). Analgesic tests occurred on Day16 and Day20. Withdrawal assessment occurred on Day21 and Day22.

Male and female mice C57BL/6J (*n* = 8/sex/group) received daily peanut butter ball (either with or without PLX) at 1300 h for 22 days. On day 14, mice received twice daily escalating doses of oxycodone (10 mg–40 mg/kg oxycodone) or saline (Veh) for 9 days prior to euthanization (Figure 1). The escalating dosing period consisted of twice daily subcutaneous injections administered at 09:00 and 17:00 h as follows: Day 14 (10 mg/kg), Day 15 (20 mg/kg), Day 16–20 (30 mg/kg), and Day 21 (40 mg/kg). A single 40 mg/kg administration (day of euthanasia) was given on Day 22. On day 21, following the 40 mg/kg oxycodone or saline injection, mice received 5 mg/kg naloxone (i.p., route) an hour later. Video was then recorded for 20 min to quantify withdrawal behaviors (12). On day 22, similarly, mice received 5 mg/kg naloxone 1 hour after the oxycodone (40 mg/kg) and ambulatory activity was measured using locomotor activity (LMA) chambers. A separate satellite group did not receive any oxycodone during the treatment. The satellite group went through the same precipitated paradigm timeline but did not go through the behavioral tests on Day 16, Day 20, Day 21, or Day 22 (Shock Flinch Test 1 (SFT1), Shock Flinch Test 2 (SFT2), naloxone injection + video recording (Nal+video), or naloxone + locomotor measurement (Nal+LMA), respectively). Brain tissue was collected shortly after the LMA test (∼2–3 h after the oxycodone injection) or at approximately the same time of day for the satellite group and used for histochemical and molecular analyses.

### Body weight, water intake, and body temperature measurement (Days 0–22)

Biotransponders (IPTT-300) were purchased from BMDS (Avidity Science) and temperatures were read using a wireless wand (BLU-8027-IMC). Biotransponders were implanted under isoflurane anesthesia a week prior to the start of PLX treatment. Body temperatures were measured at baseline and at 30 and 60 min post Oxy or Veh injections for both morning and evening administrations. Body weight (daily) and water intake (measured every 4 days using graduated cylinders fitted to the cage) were measured during the live-phase of the study.

### Withdrawal symptoms and behavioral analysis (Days 21 and 22)

Video recording: On Day 21, mice were placed in a cage with raised ceiling to capture any jumping behavior. Mice were video recorded for 20 min (ImagingSource; Model DFK22AUC03) after 5 mg/kg Naloxone and recorded using open-source software I.C Capture from The Imaging Source. Recorded videos were analyzed using VLC media player. Mice were assessed for withdrawal expressed as Wet Dog Shakes, Teeth Chattering, Paw Shakes, Jumps, and Diarrhea. The global withdrawal score was calculated using the formula: **Global withdrawal score** = jumps*0.8 + wet dog shakes*1 + diarrhea*1.5 + paw shakes*0.35 + teeth chattering*1.5 + body tremor*1.5. All analyses of video recordings were conducted with a scorer blinded to treatment conditions. A correlational analyses for jumping behavior during withdrawal was performed between two different scorers.

LMA chambers: Locomotor activity was assessed in soundproof, light-controlled locomotor activity chambers from Omnitech Electronics. On Day 22, mice were placed in the chamber immediately after receiving 5 mg/kg naloxone and their activity was recorded for 15 min. Movement time was automatically measured in the box based on horizontal and vertical light beam breaks.

### Shock flinch test (SFT; Days 16 and 20)

To assess analgesic effect and tolerance development in response to chronic repetitive oxycodone, mice were tested for flinch magnitude over a shock intensity range using force transducers to quantify flinch magnitude (24). Mice were tested in startle reactivity chambers (San Diego Instruments, SR-LAB Startle Response System) and assessed for antinociceptive responses to varying shock intensities. The program delivered 0 (background), 0.1, 0.2, or 0.4 mA shocks (Duration: 500 ms). The resulting flinch from each shock was recorded as an mV response and graphed. The program was 22 min long with eight presentations of each stimulus type. Mice were tested twice through treatment; the first test corresponded with the first 30 mg/kg Oxy (or vehicle) administration on Day 16 and the second test occurring following the last morning 30 mg/kg Oxy (or vehicle) dose on Day 20. Testing occurred 30 min following drug administration.

### Tissue collection (Day 22)

Mice were anesthetized with 2,2,2-tribromoethanol (12.5 mg/ml; dose (uL) = weight (g) x 20) then perfused with 1X PBS. Brain tissue was dissected for either immunofluorescence or cytokine analysis. The left hemisphere was collected and post-fixed at 4°C in 4% PFA for 24 h then moved to 70% ethanol until ready for slicing. Samples were submitted to the histology core at Indiana University School of Medicine to embed in paraffin then slice. Tissue was sliced at 5 microns coronally and stained using the mounted staining method. The hippocampus and cortex were crudely dissected and snap frozen from the right hemisphere for cytokine analysis.

### Immunofluorescence

The primary antibodies IBA1 (ab5076), GFAP (Invitrogen 13-0300) and the secondary antibodies Donkey anti-goat (Invitrogen A11055) and Donkey anti-rat (SouthernBiotech 6430-31) were used for immunofluorescence staining. Slides were visualized with (Leica DM6 microscope; Leica DFC 7000 GT camera) and cells were counted using the commercially available Imaris software.

### Mounted slice staining

On Day 1, slides were placed in a multi-slide carrier where they were immersed in different liquids in sequence: Formula 83 (Xylene substitute) (4x, 5 min each), 100% ethanol (2x, 10 min each), 95% ethanol (2x, 10 min each), 70% ethanol (2x, 10 min each), 50% ethanol (2x, 10 min each), distilled water (2x, 10 min each). Antigen retrieval was performed using citrate buffer in a pressure cooker (100°C for 15 min) then the slides were allowed to cool to room temperature before proceeding. The slides were then washed with distilled water (2x, 5 min each), blocked with peroxidase (15 min), and washed again with distilled water (2x, 5 min each) before blocking with 5% Donkey serum (2x, 15 min each). Slides were then incubated in primary IBA1 (1:500) of GFAP (1:1000) in 5% Donkey serum in 0.3% PBST overnight at 4°C. Day 2, slides were washed with 5% Donkey serum (2x, 10 min each) then incubated in secondary antibody (1:1000; 1 h; covered in foil from this point on). Slides were then washed with 5% Donkey serum (2x, 10 min each), allowed to airdry then cover-slipped with Prolong Gold with DAPI.

### Proinflammatory cytokine analysis

The V-PLEX proinflammatory mouse panel 1 kits (K15048D) was purchased from MSD. The kit allowed for the simultaneous measurement of 10 cytokines (IFN-γ, IL-1β, IL-2, IL-4, IL-5, IL-6, IL-10, IL-12p70, KC/GRO, TNF-α) in the same well. Cytokines were normalized to protein measured using a BCA kit (Thermo Scientific Pierce Rapid Gold BCA kit). There were no deviations from the instruction and protocol provided in either kit.

### Statistical analysis

Analysis was done using either GraphPad Prism or JMP. For GraphPad Prism, we used a *p* < 0.05 for statistical significance in any of Three-way, Two-way, Two-way repeated measure (RM), One-way, and two-tailed t-test. Male and female groups were collapsed during Tukey’s *post hoc* analyses when Sex did not interact with Treatment. JMP was used for inter-rater correlation and R-squared calculations. All statistical outputs are compiled and presented in Table 1. All data points are expressed as mean ± SEM.

**TABLE 1:** Complete statistical output for Figure 1 through Figure 7.

Figure	Statistical test	Factor	F-value	p-value	Post-hoc	p-value
2A	Two-way ANOVA	Interaction	(2, 41) = 0.13	0.874	Veh vs. Oxy	<0.0001
		Sex	(1, 41) = 1.44	0.236	Veh vs. PLX+Oxy	<0.0001
		Treatment	(2, 41) = 14.90	<0.0001	Oxy vs. PLX+Oxy	0.914
2B	Two-way ANOVA	Interaction	(2, 39) = 1.14	0.328	N/A	
		Sex	(1, 39) = 0.04	0.837		
		Treatment	(2, 39) = 0.95	0.393		
3A	Linear regression	Slope	(1, 4) = 5.87	0.072	N/A	
		Intercept	(1, 5) = 5.11	0.073		
		Equation: PLX+Oxy (y = 342.1*x+12.58)				
		Equation: Oxy (y = 583.7*x+17.16)				
3B	Linear regression	Slope	(1, 4) = 16.09	0.016	N/A	
		Equation: Oxy (y = 1024*x−3.331)				
		Equation: PLX+Oxy (y = 452.5*x+13.07)				
3C	Linear regression	Slope	(1, 4) = 8.64	0.042	N/A	
		Equation: SFT1 (y = 583.7*x+17.16)				
		Equation: SFT2 (y = 1024*x−3.331)				
3D	Linear regression	Slope	(1, 4) = 1.56	0.279	N/A	
		Intercept	(1, 5) = 2.07	0.210		
		Equation: SFT1 (y = 342.1*x+12.58)				
		Equation: SFT2 (y = 452.5*x+13.07)				
3E	Linear regression	Slope	(1, 4) = 1.99	0.230		
		Intercept	(1, 5) = 6.90	0.047		
		Equation: SFT1 (y = 6698*x+221.5)				
		Equation: SFT2 (y = 5039*x+12.18)				
4A	Two-way ANOVA	Interaction	(2, 37) = 0.46	0.631	N/A	
		Sex	(1, 37) = 30.24	<0.0001		
		Treatment	(2, 37) = 0.17	0.843		
4B	Two-way ANOVA	Interaction	(2, 37) = 3.19	0.052	Veh vs. Oxy	<0.0001
		Sex	(1, 37) = 3.44	0.071	Veh vs. PLX+Oxy	<0.0001
		Treatment	(2, 37) = 20.29	<0.0001	Oxy vs. PLX+Oxy	0.929
4C	Two-way ANOVA	Interaction	(2, 37) = 0.03	0.964	Veh vs. Oxy	<0.0001
		Treatment	(2, 37) = 24.89	<0.0001	Veh vs. PLX+Oxy	<0.0001
		Sex	(1, 37) = 0.45	0.503	Oxy vs. PLX+Oxy	0.817
4D	Three-way ANOVA	Treatment	(2, 36) = 38.61	<0.0001		
		Sex	(1, 36) = 2.26	0.140		
		Time	(1, 36) = 23.55	<0.0001		
		Treatment X Sex	(2, 36) = 1.03	0.366		
		Treatment X Time	(2, 36) = 3.22	0.051		
		Sex X Time	(1, 36) = 0.150	0.700		
		Treatment X Sex X Time	(2, 36) = 1.93	0.159		
	Two-way ANOVA RM	Interaction	(2, 39) = 3.57	0.0376	Day 16	
		Time	(1, 39) = 24.50	<0.0001	Veh vs. Oxy	<0.0001
		Treatment	(2, 39) = 37.09	<0.0001	Veh vs. PLX+Oxy	<0.0001
		Subject	(39, 39) = 2.60	0.001	Oxy vs. PLX+Oxy	0.9995
					Day 20	
					Veh vs. Oxy	<0.0001
					Veh vs. PLX+Oxy	<0.0001
					Oxy vs. PLX+Oxy	0.597
					Day 16 vs. 20	
					Veh	0.757
					Oxy	0.0001
					PLX+Oxy	0.013
5A	Two-way ANOVA	Interaction	(2, 42) = 1.42	0.252	Veh vs. Oxy	<0.0001
		Sex	(1, 42) = 3.89	0.055	Veh Vs. PLX+Oxy	<0.0001
		Treatment	(2, 42) = 39.09	<0.0001	Oxy vs. PLX+Oxy	0.713
5B	Two-way ANOVA	Interaction	(2, 42) = 1.732	0.189	Veh vs. Oxy	<0.0001
		Sex	(1, 42) = 7.375	0.009	Veh vs. PLX+Oxy	<0.0001
		Treatment	(2, 42) = 100.8	<0.0001	Oxy vs. PLX+Oxy	0.907
5C	Two-way ANOVA	Interaction	(2, 42) = 2.22	0.121	Veh vs. Oxy	<0.0001
		Sex	(1, 42) = 1.67	0.202	Veh vs. PLX+Oxy	<0.0001
		Treatment	(2, 42) = 22.39	<0.0001	Oxy vs. PLX+Oxy	0.87
6A	Two-way ANOVA	Interaction	(2, 42) = 1.19	0.313	Veh vs. Oxy	0.264
		Sex	(1, 42) = 3.67	0.061	Veh vs. PLX+Oxy	<0.0001
		Treatment	(2, 42) = 48.59	<0.0001	Oxy vs. PLX+Oxy	<0.0001
6B	Two-way ANOVA	Interaction	(2, 42) = 0.28	0.755	N/A	
		Sex	(1, 42) = 2.71	0.107		
		Treatment	(2, 42) = 0.75	0.476		
7A	Two-way ANOVA	Interaction	(2, 42) = 0.20	0.813	N/A	
		Sex	(1, 42) = 9.84	0.003		
		Treatment	(2, 42) = 0.32	0.727		
7B	Two-way ANOVA	Interaction	(2, 42) = 0.59	0.553	N/A	
		Sex	(1, 42) = 1.17	0.284		
		Treatment	(2, 42) = 1.87	0.166		
7C	Two-way ANOVA	Interaction	(2, 42) = 0.41	0.663	Veh vs. Oxy	0.239
		Sex	(1, 42) = 5.88	0.019	Veh vs. PLX+Oxy	0.0009
		Treatment	(2, 42) = 7.74	0.001	Oxy vs. PLX+Oxy	0.070
7D	Two-way ANOVA	Interaction	(2, 42) = 0.75	0.477	N/A	
		Sex	(1, 42) = 14.21	0.0005		
		Treatment	(2, 42) = 1.30	0.282		
7E	Two-way ANOVA	Interaction	(2, 42) = 0.67	0.517	N/A	
		Sex	(1, 42) = 11.77	0.001		
		Treatment	(2, 42) = 0.79	0.457		
7F	Two-way ANOVA	Interaction	(2, 42) = 0.06	0.939	N/A	
		Sex	(1, 42) = 1.90	0.175		
		Treatment	(2, 42) = 2.36	0.106		

## Results

### PLX treatment did not affect oxycodone-induced weight loss or treatment-induced decrease in water intake

**FIGURE 2:**
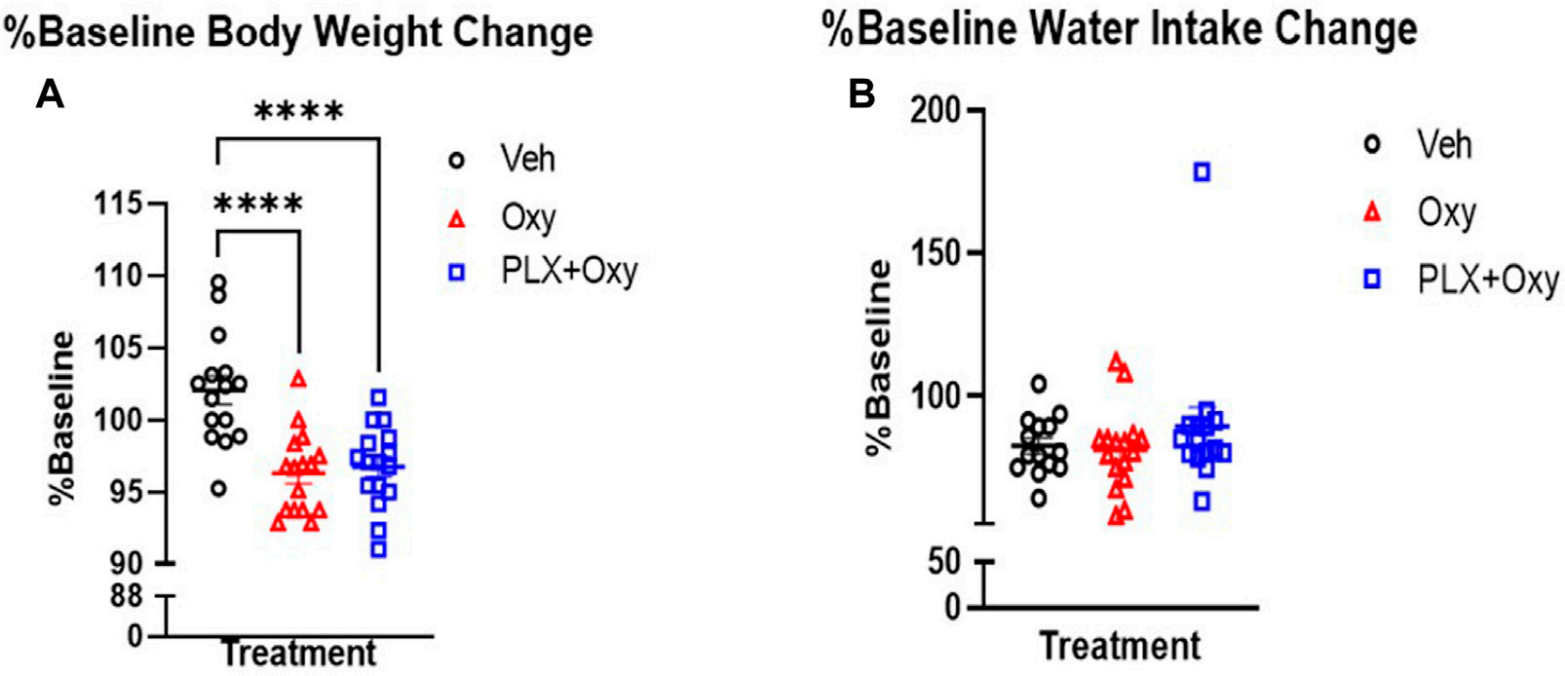
Change in body weight and water intake throughout PLX and Oxy treatment. Mice were weighed and their water intake recorded every fourth day during the PLX treatment alone (No Oxy) and used as baseline to evaluate change on the day of euthanization (Day 22) at the end of the Oxycodone regimen. **(A)** Oxycodone decreased body weight (%Baseline) by the end of the treatment and PLX had no effect. (*n* = 16/group). **(B)** Neither oxycodone or PLX had an effect on water intake. Overall, all groups decreased their water intake with time (*n* = 16/group) ****: *p* < 0.0001. Unless indicated with an asterisk, groups were compared and were not significantly different.

Body weights of male and female mice were measured every 3 days through PLX treatment alone (Baseline) and daily during the PLX and Oxy treatment period. Due to intrinsic differences in average body weight between the sexes, the data was transformed into % Baseline (body weight on each post-Oxy treatment day/averaged 3 days of last body weights prior to beginning of treatment regimen) to normalize body weights for each sex to better allow for analysis of the effects of Treatment across Sex (raw data in Supplementary Figures S1A,B). In looking at the last Oxy treatment day of 40 mg/kg, only a main effect of Treatment was observed, where both Oxy and PLX groups had reduced weight gain over the treatment period. However, Oxy and PLX+Oxy groups did not differ from one another (Figure 2A; Table 1).

We also measured water intake throughout the treatment. In similarity to body weight, there was a significant intrinsic difference in baseline water intake between male and female mice. We therefore calculated the %Baseline change on euthanization day. A total of three baseline measurements (collected every fourth day during the PLX alone period prior to any Oxycodone) were averaged for each treatment and each sex and compared with fluid intake on the last day (euthanization day; Day 22) and the %Baseline was graphed. The complete dataset is presented in Supplementary Figures S1C,D. A two-way ANOVA showed a lack of sex or treatment effect (Figure 2B; Table 1). Overall, we observed the often reported decrease in body weight with Oxy treatment, but concurrent PLX treatment did not restore these losses. In contrast, water intake consistently decreased across all groups implying a non-specific effect on mice such as daily handling and injections.

### PLX treatment prevents the development of analgesic tolerance

To quantify the antinociceptive effects using the shock-flinch test, we used a linear regression model and compared slopes of flinch magnitude over increasing shock intensities (0, 0.1, 0.2, and 0.4 mA). A steeper slope value indicates lower analgesic response (i.e., greater flinch responses with increased shock intensity) compared to a smaller slope value. If the slope was not significantly different, our follow-up test compared the intercepts for statistical difference. Statistically different intercepts indicate a change in analgesia with the larger intercept value indicating lower analgesic response (two lines that are parallel but one is higher on the y-axis). Sex was first analyzed as a factor and was not significant (see Supplementary Figures S2A–F), so data were collapsed across sex to simplify data analysis.

**FIGURE 3:**
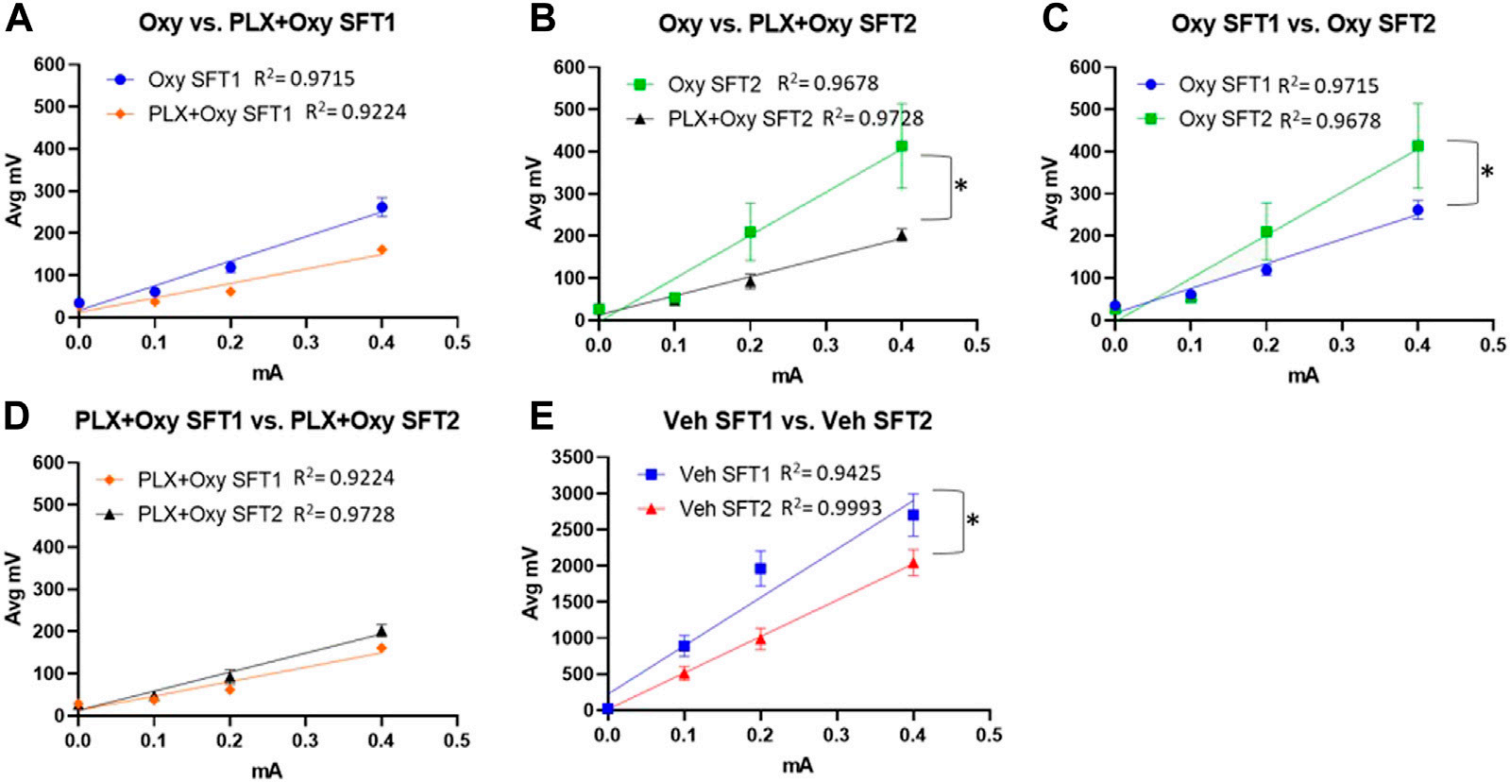
Results of the analgesic tests SFT1 (Day16) and SFT2 (Day20). Mice were treated with 30 mg/kg Oxycodone on Day 16 and Day 20 and subsequently placed in Shock Flinch Test chambers 30 min after injection to measure response to 0, 0.1, 0.2, and 0.4 mA shocks. **(A)** Avg mV response to each stimulus intensity after the first 30 mg/kg oxycodone challenge (SFT1). Responses in PLX+Oxy mice were not significantly different than Oxy alone (*n* = 16/point). **(B)** Avg mV response to each stimulus intensity after the last 30 mg/kg dose (SFT2) Oxy and PLX+Oxy treatments were significantly different during the second SFT test (*n* = 16/group). **(C)** Avg mV response to each stimulus intensity in Oxy groups between SFT1 and SFT2 was significantly different (*n* = 15–16/group) **(D)** Avg mV response to each stimulus intensity in PLX+Oxy groups between SFT1 and SFT2 was not significantly (*n* = 16/group) **(E)** Avg mV response to each stimulus intensity in Veh groups significantly differed between SFT1 and SFT2 (*n* = 16/group). Unless indicated with an asterisk, groups were compared and were not significantly different.

The high dose of Oxy used (30 mg/kg) resulted in a 91%–94% reduction of the flinch response compared to Veh, which reduced the sensitivity in detecting Oxy vs. PLX+Oxy treatment differences. Thus, we directly assessed whether Oxy and PLX+Oxy groups differed in each SFT. Treatment did not differ significantly in SFT1, although a trend existed for the PLX+Oxy group to exhibit greater Oxy-induced analgesia (Figure 3A; Table 1). In assessing Treatment during SFT2, the PLX+Oxy group showed enhanced analgesia (i.e., smaller flinch response) compared to Oxy alone (Figure 3B; Table 1). To further evaluate tolerance, the change between SFT1 and SFT2 for each treatment was analyzed. In the Oxy group, the SFT2 slope was significantly steeper than SFT1 indicating tolerance (i.e., less nociceptive analgesia (Figure 3C; Table 1). In contrast, no differences between SFT2 and SFT1 existed in the PLX+Oxy group (Figure 3D; Table 1). Figure 3E shows a significant difference between SFT2 and SFT1 in the Veh group (no Oxy given) in the Intercept, indicating that startle reactivity generally was lower on the second test, possibly reflecting habituation processes (Figure 3E; Table 1). Collectively, the analgesic response was somewhat blunted in the Oxy but not the PLX+Oxy group and behavioral tolerance was not observed with PLX treatment.

### PLX treatment does not affect oxycodone-induced hypothermia

**FIGURE 4:**
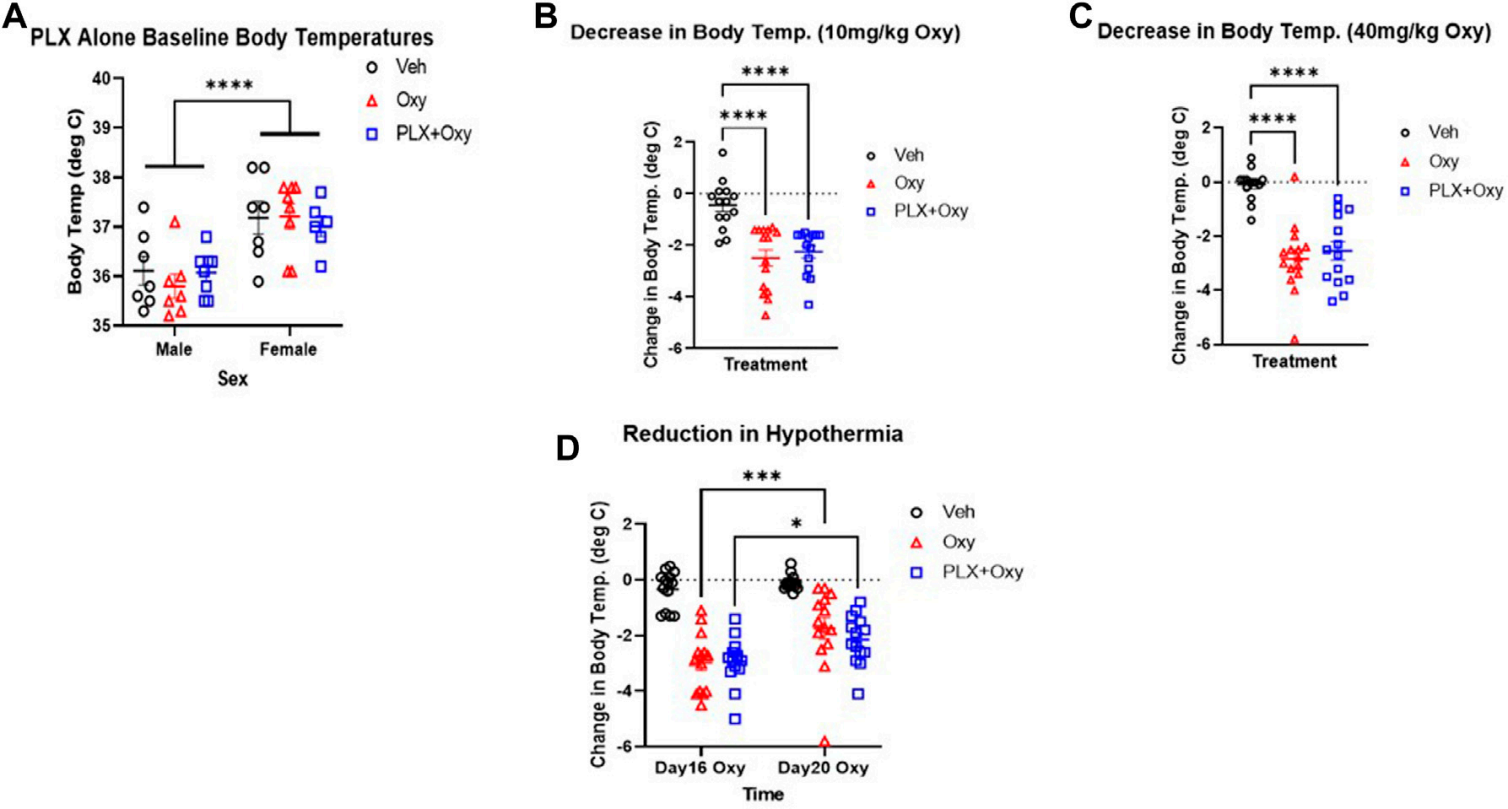
Hypothermia in mice in response to Oxy injections on different days and doses. Body temperature was measured the morning of Day14 just before the first Oxy injection in the paradigm during the **(A)** PLX alone period (which reflects 14 days of regular peanut butter balls (Veh and Oxy groups) and PLX peanut butter balls (PLX+Oxy group) treatment **(B–D)** Body temperatures were measured at 30 and 60 min after Veh or Oxy injections and the greater hypothermic response of the two was used in calculating change from baseline. **(A)** PLX did not affect baseline body temperatures but there was a sex effect (*n* = 8/group) **(B,C)** Oxycodone-induced hypothermia (change from baseline) was not reversed by PLX at either 10 mg/kg dose (*n* = 14–16/group) or 40 mg/kg dose (*n* = 14–16/group) **(D)** PLX had no effect on the extent of hypothermia on Day 16 vs. Day 20, however, oxycodone and PLX+Oxy group oxycodone-induced hypothermia decreased in Day 20 compared to Day 16 (*n* = 14–16/group). ***: *p* = 0.0001 *: *p* = 0.01. ***: *p* < 0.0001. Unless indicated with an asterisk, groups were compared and were not significantly different.

Opioids are known to dysregulate body temperature which is related to their autonomic suppression effects (16, 25, 26, 27). First, baseline body temperatures were analyzed between the groups during the PLX treatment to determine if PLX had an effect on temperature prior to the Oxy treatment regimen. For this analysis, Veh (plain peanut butter) *versus* PLX (PLX in peanut butter) group body temperatures were measured just prior to the first Oxy administration (morning of Day 14). Analysis revealed only a main effect of Sex (Figure 4A; Table 1). On average, females had a higher baseline temperature compared to male and PLX treatment did not impact basal body temperature levels.

Next, analyses of the first dose of Oxy (10 mg/kg) and first dose of 40 mg/kg Oxy were conducted to determine whether PLX treatment affected initial or chronic treatment effects of Oxy-induced hypothermia. Data were transformed to delta scores using the following formula: (peak hypothermia at either 30 or 60 min post-Oxy dose – baseline body temperature prior to dosing). Analysis of the initial 10 mg/kg Oxy dose revealed main effects of Treatment but not Sex. Both Oxy and PLX+Oxy groups differed from Veh (but not each other) and exhibited a decrease of ∼2.5°C. (Figure 4B; Table 1). Of note, the Treatment X Sex interaction approached significance and was comprised of Oxy and PLX+Oxy female groups exhibiting ∼1°C greater decrease in body temperature compared to males. Following the Oxy treatment regimen, body temperatures were taken following the first dose of 40 mg/kg. A main effect of Treatment was found and comprised of Oxy and PLX+Oxy groups differing from Veh, but not each other, exhibiting approximately a 3°C hypothermic response (Figure 4C; Table 1). Lastly, post-naloxone body temperatures were taken, while vehicle significantly differed from Oxy treated animals, Oxy and PLX+Oxy groups did not differ (data not shown). Overall, these data show robust Oxy-induced hypothermia that was unaffected by chronic PLX treatment.

### PLX treatment does not attenuate tolerance of oxycodone-induced hypothermia after repetitive administration

To directly assess tolerance to the hypothermia effect of Oxy (28), we analyzed change in body temperature during the evening dose of the first 30 mg/kg dose day (Day 16) and last 30 mg/kg dose day (Day 20). We chose the evening doses because the morning injections coincided with behavioral testing. There were Time (Day 16 vs. Day 20) and Treatment main effects in the Three-way ANOVA. Collapsing across sex, main effects of Treatment and Time were observed as well as a significant interaction between these factors in the Two-way ANOVA repeated measures (Figure 4D; Table 1). The source of the interaction was both Oxy and PLX+Oxy Day 20 hypothermic responses being significantly less than the Day 16 response, indicating tolerance. Veh group temperatures did not differ across Time, nor did Oxy and PLX+Oxy groups differ from each other (Figure 4D; Table 1). These data demonstrate the development of hypothermic tolerance to oxycodone treatment, but that PLX treatment did not impact neither the amplitude of Oxy-induced hypothermia nor tolerance development with repeated dosing.

### PLX treatment did not affect naloxone-precipitated withdrawal symptoms or locomotion in oxycodone-treated mice

**FIGURE 5:**
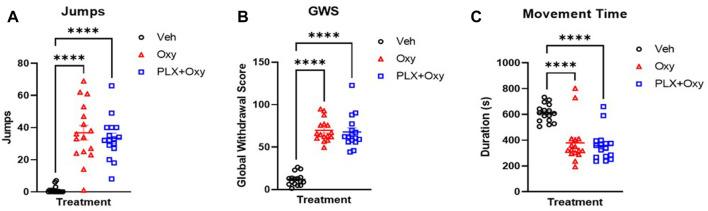
Naloxone-induced withdrawal behavior in oxycodone-dependent and non-dependent mice. Jumps, withdrawal symptoms, and movement time were measured on either Day 20 **(A,B)** or Day 22 **(C)** in response to a Naloxone challenge 1 h after receiving a 40 mg/kg Oxy or Veh injection. **(A)** Male and female (sex collapsed) number of jumps in 20 min after oxycodone (40 mg/kg) then Naloxone (5 mg/kg) challenge. Oxycodone treated mice increased jumping during withdrawal, but PLX treatment had no effect (*n* = 16/group). **(B)** Male and female Global Withdrawal Score (GWS) (sex collapsed) in 20 min after oxycodone (40 mg/kg) then Naloxone (5 mg/kg) challenge. PLX treatment did not decrease oxycodone-induced increase in GWS (*n* = 16/group). **(C)** Movement time in chamber during 15 min after oxycodone (40 mg/kg) then Naloxone (5 mg/kg) (*n* = 16/group). Withdrawal from oxycodone decreased movement time, PLX treatment had no effect. ****: *p* < 0.0001. Unless indicated with an asterisk, groups were compared and were not significantly different.

We utilized both observational and automated approaches to assess withdrawal symptoms through naloxone-precipitated withdrawal. On Day 21, Oxy-treated mice received 40 mg/kg oxycodone and 1 h after challenged with 5 mg/kg naloxone. Vehicle controls also received naloxone. Mice were video recorded for 20 min and assessed for withdrawal symptoms (jumps, teeth chattering, diarrhea, paw shake, wet dog shakes, body tremors). A reliable withdrawal indicator is the number of jumps during withdrawal. There was a main effect of Treatment, but no effects of Sex or an interaction between these factors. Due to a lack of sex effect, we collapsed the sexes and found a difference between Oxy and PLX+Oxy vs. Veh (Figure 5A; Table 1). Inter-rater correlation analysis yielded an R-squared of 0.985 (Supplementary Figure S3). In addition, a Global Withdrawal Score (GWS) was calculated to include other withdrawal symptoms and account for mice that innately exhibit withdrawal symptoms other than jumping. In contrast to number of jumps, there was a sex and treatment but not an interaction effect in GWS. On average, females had a higher GWS in both Oxy and PLX+Oxy groups compared to males however the treatments did not differ in either sex. The sexes were collapsed to show a difference between Veh vs. Oxy treated animals with PLX having no effect (Figure 5B; Table 1).

On Day 22, mice again received Oxy or vehicle, administered naloxone as described above, and were assessed for 15 min of open-field locomotor activity using total movement time as the primary measure. There was a main effect of Treatment, but not Sex or the interaction of these factors. Post-hoc analysis determined that both Oxy alone and PLX + Oxy groups differed from Veh (*p* < 0.0001), but not from each other, *p* = 0.870; Figure 5C). In summary, PLX treatment did not influence expression of naloxone-induced opioid withdrawal.

### PLX treatment decreased IBA1+ cell counts with no effect on GFAP+ cells in hippocampus

**FIGURE 6:**
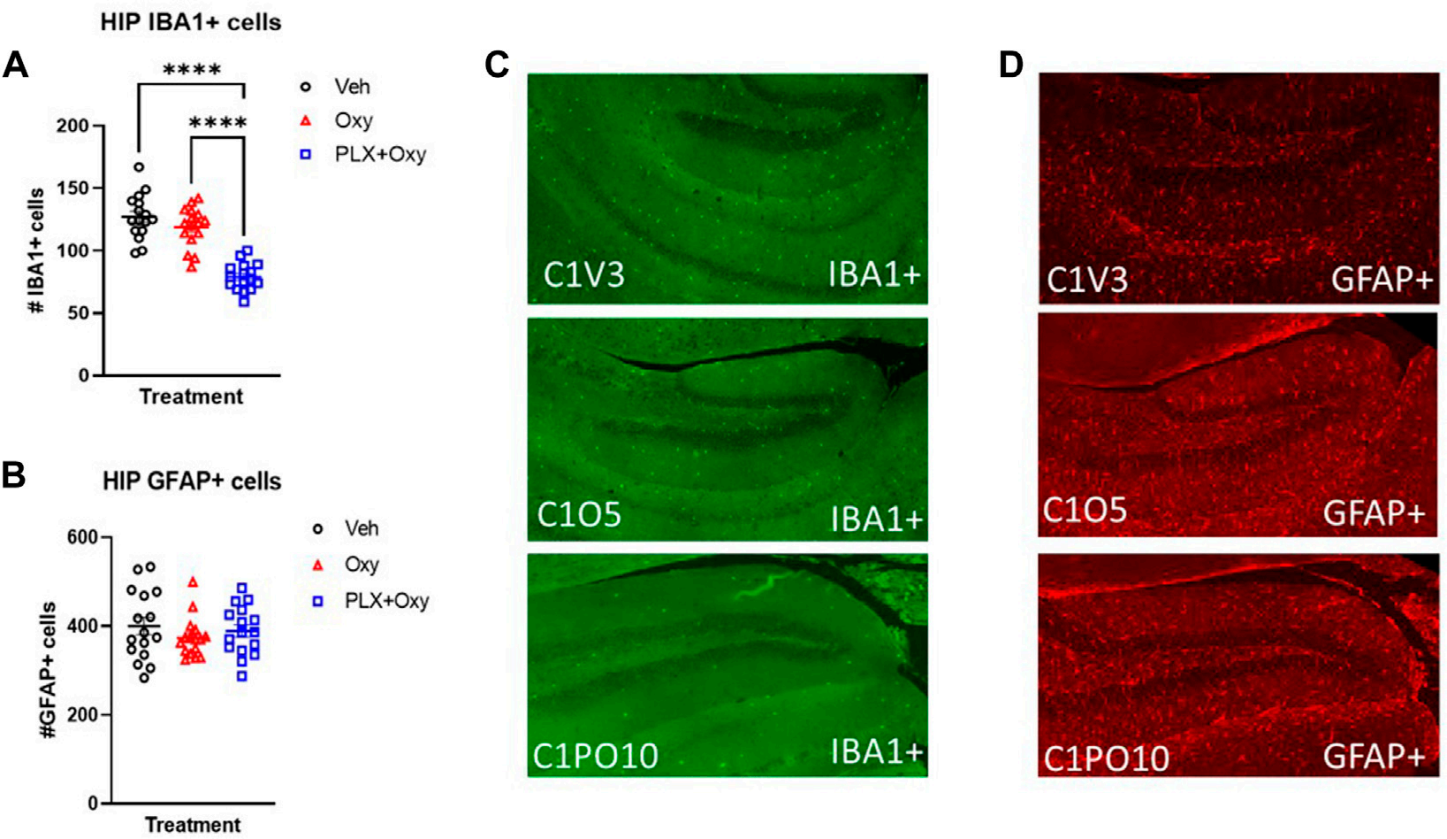
Glial activity markers in oxycodone-dependent or non-dependent mice. IBA1+ and GFAP+ stained hippocampus tissue collected from mice after euthanization on Day 22 (tissue collection occurred 2–3 h after Oxy or Veh injection; mice received 40 mg/kg Oxy or Veh, 1 h later received Naloxone and euthanized half an hour after Naloxone). **(A)** Male and female IBA1+ cell counts in the hippocampus were collapsed across sexes for each treatment to show ∼40% microglial reduction (*n* = 16/group). ***: *p* < 0.0001 **(B)** Male and female GFAP cell counts in the hippocampus were collapsed across sexes for each treatment and microglial reduction had no effect on GFAP expression. **(C,D)** Representative IHC staining for IBA1 and GFAP (*n* = 16/group). C1V = Veh, C1O = Oxy, C1PO = PLX+Oxy. Unless indicated with an asterisk, groups were compared and were not significantly different.

We confirmed that PLX treatment successfully knocked down microglial expression. Quantification of IBA1+ cells in the hippocampus using IHC methodology showed that PLX effectively knocked down microglia expression. Analysis revealed only a main effect of Treatment. The PLX treatment regimen reduced microglia expression by ∼40% in the PLX+Oxy group compared to the Oxy and Veh. Interestingly and in contrast to other reports with morphine (28, 29), chronic Oxy did not affect microglia levels compared to Veh controls (Figures 6A,C; Table 1). Because neuronal microenvironment is maintained by multiple glial cells including astrocytes, astrocyte levels were quantified. Neither Oxy nor PLX altered the number of GFAP+ cells compared to Veh (Figures 6B,D; Table 1). In order to account for PLX specific effects, a separate satellite PLX3397 control group (i.e., no Oxy treatment) was run simultaneously with the Oxy treatment experiment, but tissue collection occurred in the days shortly after the end of the precipitated withdrawal experiment tissue collection. In agreement with the PLX+Oxy group in the present experiment, PLX suppressed IBA1+ cells by similar levels (Supplementary Figure S4A) without affecting expression levels of GFAP+ cells (Supplementary Figure S4B). Due to tissue collection and slicing method, tissue quality did not permit staining in the somatosensory cortex, as a result, IHC for the cortex was not performed. In summary, PLX treatment significantly reduced microglia levels by approximately 40% and Oxy treatment did not affect either Veh or PLX levels. There was no compensatory increase in astrocyte expression.

### PLX treatment synergistically increases KC/GRO, male and female mice differentially express IL-5 and IL-6 irrespective of treatment

Cytokines are strong signaling mediators between cells. Chronic intrathecal administration of morphine has been found to elevate various cytokine and chemokines in rat CSF and spinal tissue (3, 14). However, changes in cytokines and chemokines from opioid dependence specifically during acute withdrawal have not been investigated with oxycodone. We sought to understand whether oxycodone dependence and subsequent withdrawal results in alterations in cytokine levels. Furthermore, we sought to understand whether knocking down microglia, potent cytokine producing immune cells, would have an effect on cytokine and chemokine levels during withdrawal from opioids. We utilized a commercially available cytokine assay to evaluate the following cytokines: IFN-γ, IL-1β, IL-2, IL-4, IL-5, IL-6, IL-10, IL-12p70, KC/GRO, TNF-α in either the somatosensory cortex or hippocampus to determine if there are any regional differences. While most of the cytokines were below our detectable limit (IFN-γ, IL-1β, IL-2, IL-4, IL-10, IL-12p70, TNF-α), IL-5, IL-6, and KC/GRO were detectable and quantifiable.

**FIGURE 7:**
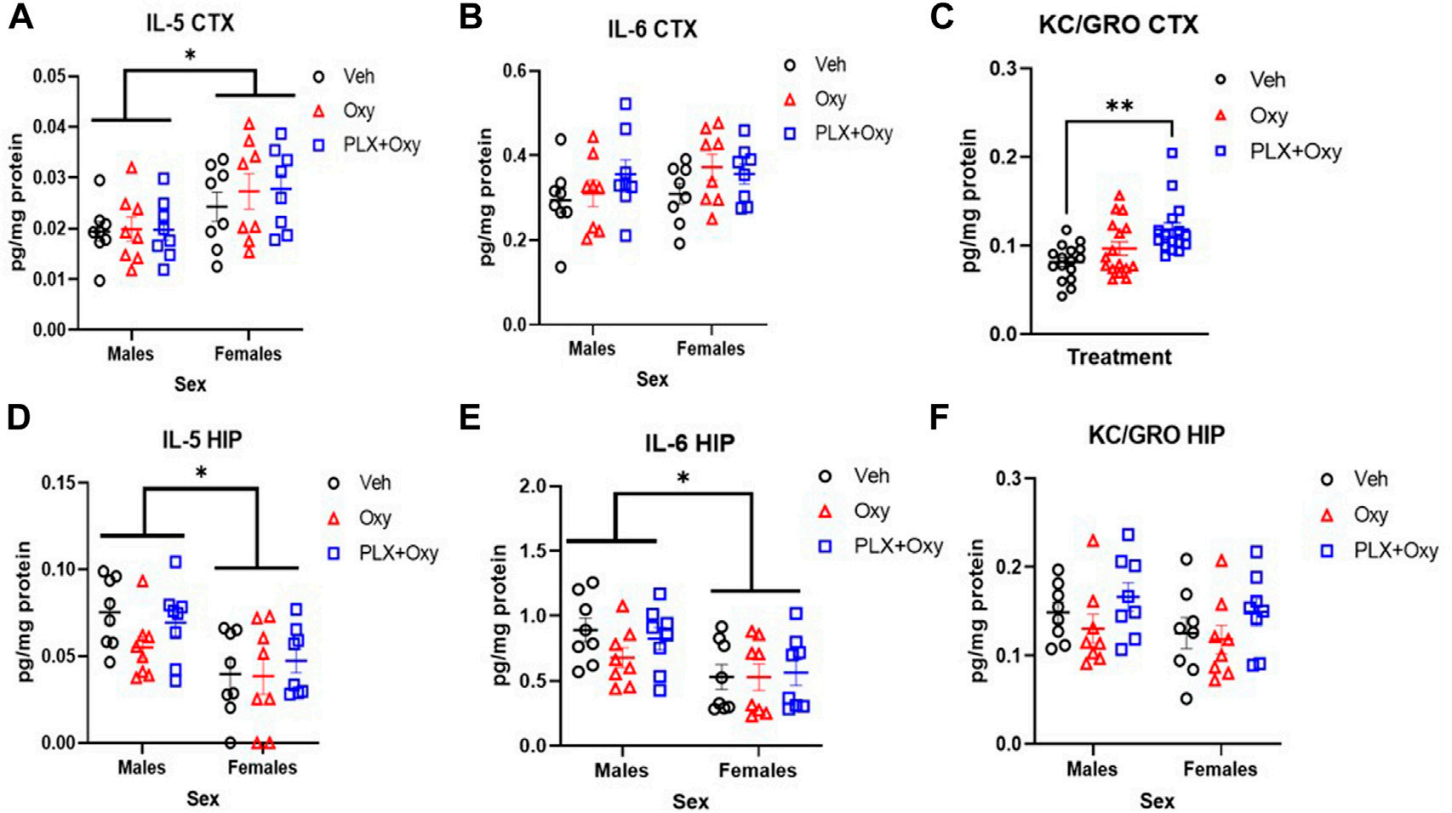
Cytokine analysis from hippocampus (HIP) and cortex (CTX) in oxycodone-dependent and non-dependent mice. Cortex and hippocampus tissue were collected from mice on Day 22 (tissue collection occurred 2–3 h after Oxy or Veh injection; mice received 40 mg/kg Oxy or Veh, 1 h later received Naloxone and euthanized half an hour after Naloxone). **(A)** While Males and females differed significantly in IL-5 expression in the cortex, neither oxycodone nor PLX had an effect *p = 0.003 **(B)** There were no sex or treatment effect on the expression of IL-6 in the cortex **(C)** Oxycodone treated-PLX-treated mice express significantly higher KC/GRO compared to vehicle. **p = 0.0009 **(D)** Males and females differ significantly in IL-5 expression in the hippocampus however oxycodone and PLX had no effect *: *p* = 0.0005 **(E)** Males and females differed significantly in their expression of IL-6 in the hippocampus however neither drug had an effect *: *p* = 0.001 **(F)** There were no treatment or sex effects in KC/GRO expression in the hippocampus (*n* = 16/group for each graph). Unless indicated with an asterisk, groups were compared and were not significantly different.

In the cortex, only a main effect of Sex was observed for IL-5 expression, with females having higher levels than males (Figure 7A; Table 1). In contrast, there were no significant differences with IL-6 (Figure 7B; Table 1). However, KC/GRO expression was both Sex and Treatment dependent, but did not interact with one another. Overall, females had higher KC/GRO values than males. Collapsing across sexes in our *post hoc* test, the PLX+Oxy group expressed significantly more KC/GRO than Veh, but Oxy vs. Veh groups were not significantly different (Veh vs. PLX+Oxy, *p* = 0.0009; Veh vs. Oxy, *p* = 0.239). Interestingly, higher levels of KC/GRO in the PLX+Oxy group approached significance against the Oxy group (*p* = 0.070) (Figure 7C; Table 1).

In the hippocampus, similar effects were found with IL-5 and IL-6. We found only a main effect of Sex in IL-5, with male IL-5 levels greater than females. (Figure 7D; Table 1). Similarly for IL-6 expression, males expressed higher levels than females regardless of treatment (Figure 7E; Table 1). Unlike the cortex, no significant differences in KC/GRO existed (Figure 7F; Table 1). As for the control group, none of the quantifiable cytokines (IL-5, IL-6, KC/GRO) were different in Veh vs. PLX in either the cortex or hippocampus (Supplementary Figure S5).

## Discussion

The major findings of this study were that a microglial reduction of approximately 40% did not affect many acute responses to Oxy nor the magnitude of naloxone-precipitated withdrawal in male and female C57BL/6J mice. In measures of tolerance, oxycodone-induced hypothermia was unaffected by microglial reduction and repeat doses decreased hypothermia similarly in both Oxy and PLX+Oxy groups. However, the partial knock-down of microglia expression did have physiological consequences. In measuring nociceptive responses in the shock-flinch test, PLX+Oxy mice tended to have less flinch response to shock with the first dose of 30 mg/kg and exhibited significantly reduced tolerance over 4 days of administration at this dose. Of the three quantifiable cytokines, we observed differences in either a sex- or treatment-dependent manner. The most robust neuroinflammatory response concerned increased KC/GRO expression in the cortex that was greatest in the PLX+Oxy treatment group. However, we did not find a difference between treatments or sex in cortical IL-6 or hippocampal KC/GRO. Only sex-dependent expression changes were found for IL-5 and IL-6. Independent of treatment, males expressed significantly less IL-5 in the cortex, but significantly increased IL-5 and IL-6 in the hippocampus compared to females. Finally, microglial depletion did not produce a compensatory change in astrocyte levels as determined by GFAP staining.

Despite efforts to curb opioid use, they remain the most effective treatment for most pain states. Chronic use, however, leads to rapid development of tolerance and eventual dependence. In opioid-dependent individuals, abrupt cessation of opioid use results in distressful and potentially dangerous acute withdrawal symptoms that contribute to the addiction relapse cycle. The neural circuitry that mediates acute and chronic effects of opioids have been investigated extensively over the years. Glial contribution is not surprising considering astrocytes and microglia express opioid receptors and can regulate inflammatory signaling, oxidative stress response, and neurotransmitter balance in the neuron’s microenvironment (30, 31, 32). Furthermore, opioid dependence and pain states have some distinct pathologies and associated brain regions but also overlap in multiple brain regions important to each (5, 33, 34, 35). We highlight two of the overlapping brain regions and attempt to describe the inflammatory profile in the somatosensory cortex and hippocampus. The somatosensory cortex is central to pain signaling and densely populated by opioid receptors (30, 34, 36, 37). Similarly, in addition to its critical role in cue- and context-associated drug relapse, particularly in protracted withdrawal, the hippocampus integrates immediate pain processing and some acute opioid withdrawal symptoms (13, 17, 38, 39). These data address a gap in our understanding of the role of microglial in mediating various physiological and behavioral endpoints over an opioid dependence induction regimen. In contrast to other reports, our central hypothesis that microglial depletion would alter acute opioid withdrawal intensity was not supported (12). The reason for these differences are unclear, but other studies used non-specific glial inhibitors such as minocycline (believed to inhibit microglial activity) or ibudilast, (AV411; a general anti-inflammatory agent). Yet others used an opioid receptor-specific knockout models or different mu opioid agonists. These differences may account for our different results or imply off-target effect (12, 40). However, in agreement with literature, we report a glial role, specifically microglia, in regulating opioid analgesia. This lends support to literature describing the reduction/reversal of analgesic tolerance by targeting glial activity (e.g., AV411) or antagonizing inflammation-associated cytokines, chemokines, and receptors (14, 41, 42). Importantly, these data indicate a direct role of microglia on affecting analgesic tolerance.

As mentioned, cytokines have been implicated in the development of analgesic tolerance. Various cytokines regulate the expression and/or conductance of neuronal ligand-gated ion channels. For instance, major inflammatory cytokines such as TNFα and IL-1β modulate glutamate receptor (NMDA/AMPA) conductance and/or surface expression resulting in changes in neuronal excitability (42). In parallel, cytokine-induced glutamate transporter (e.g., GLT-1, GLAST) downregulation by opioid administration can contribute to tolerance by increasing synaptic glutamate and modulating neuronal excitability (6, 30). The immunoregulatory aspect of opioids is supported by a multitude of publications describing changes in various cytokines and chemokines in response to opioid administration particularly with morphine (3, 14). In contrast, literature on the modulation of cytokines with oxycodone treatment alone is scarce. However, changes in major cytokines (e.g., IL-1β and TNF-α) have been reported in the presence of strong proinflammatory stimuli (lipopolysaccharide) or in an already existing inflammatory disease state (43, 44, 45). Unfortunately, we were unable to detect IFN-γ, IL-1β, IL-2, IL-4, IL-10, IL-12p70, and TNF-α expression with our methods. Therefore, it is not clear whether oxycodone and/or microglial reduction altered the expression of these cytokines. Considering the critical role of cytokines in neuronal signaling, follow-up experiments utilizing more sensitive/alternative methods (western blot, qPCR, etc.) would help uncover potential players. These studies revealed differential expression of cytokines between sexes that underscore the importance of including sex as an experimental factor. Interestingly, in expanding the search into other cytokines and chemokines, we find that KC/GRO expression increases in a synergistic fashion. One other paper found changes in KC/GRO expression in the CNS in response to opioids, but it was limited to the spinal cord tissue/CSF (14). Furthermore, previous studies in our lab showed a similar increase in KC/GRO from whole-hemisphere homogenate in oxycodone-dependent mice at 24 h after the last dose (Supplementary Figure S6). This highlights the presence of other underexplored chemokines and raises the question of how they could potentially contribute to analgesia.

KC/GRO is a neutrophil chemoattractant that binds to the receptor CXCR2 (46). It has been associated with both pathological and inflammation-dependent resolution of brain injury mediated primarily by neutrophil activity (47, 48). We then predict that the source of KC/GRO is from non-neuronal CNS cells (astrocytes/endothelial cells), this is supported by literature showing increases in many CXCR2 cytokines: CXCL1 (KC/GRO), CXCL2, and CXCL8 (49). Therefore, we anticipate CXCR2 expressing neurons would likely be the target of these ligands (49). Paradoxically, CXCR2 activation on neurons is typically detrimental resulting in cell death; however, neuroprotective properties of KC/GRO/CXCR2 have been reported as well (50, 51). As a result, KC/GRO signaling is unlikely to be mediated through CNS (neuronal) CXCR2 receptors as one would conclude neuronal apoptosis and mice in our PLX+Oxy group did not exhibit pathological signs, or any health concerns compared to the Oxy alone. Alternatively, KC/GRO release could recruit peripheral neutrophils and thereby affect CNS microenvironment. Interestingly, infiltrating neutrophils have been reported to regulate pain signaling through the release of opioid peptides (52, 53). Therefore, we hypothesize that CNS KC/GRO release delays the development of oxycodone-induced analgesic tolerance. It would be interesting to follow-up with experiments addressing this hypothesis by using, for example, Cxcr2−/− mice, CXCR2 antagonist NVP CXCR2 20, or neutrophil depletion (for example anti-Ly6G) to manipulate the KC/GRO/CXCR2 signaling pathway (49, 54, 55). Overall, this an interesting finding that warrants further exploration. Targeting KC/GRO receptors systemically or locally in the CNS could uncover potential therapeutic targets.

Lastly, it is thought that hypothermia in rodents is mediated by a combination of mu, kappa, and delta opioid receptor subtypes (16, 56). This is complicated further by evidence suggesting thermoregulation is mediated differentially by different opioids at different doses (25–27). We did not see a difference in hypothermia in response to oxycodone with microglial reduction nor did we see a differential rate in the reduction of hypothermic response. It is therefore possible that body temperature is uncoupled from microglial activity in our case. This implies astrocyte/neuron dependent mechanisms of hypothermia and hypothermic tolerance that warrants further investigation.

This is work is not without its limitations. We succeeded in reducing microglial expression (∼40%) in a physiologically relevant amount that altered behavioral and neuroinflammatory responses to repeated Oxy treatment. However, whether we observe changes in withdrawal symptoms severity under greater microglial depletion levels is unknown. To this end, we have unpublished pilot data showing that >90% microglial depletion did not affect acute spontaneous withdrawal symptoms in mice treated with a similar escalating Oxy regimen. As for our molecular detection methods, we relied on the commonly used microglial marker IBA1. We did not completely account for other cells of similar lineage that also express IBA1 (peripheral macrophage). Therefore, our IBA1 staining best describes microglia/infiltrating macrophage cells present in the brain. Because robust withdrawal symptoms were not observed, we moved to a naloxone-precipitated withdrawal paradigm in the present study. Another limitation of this work is that molecular investigations were limited to cortex and hippocampus. As KC/GRO changes were only observed in cortex, it is reasonable to anticipate novel findings in other brain regions that mediate various aspects of opioid dependence, analgesia, and withdrawal (57). Furthermore, while we hypothesize that KC/GRO is released from astrocytes/neurons as mentioned above, this is yet to be verified with follow-up experiments. In addition, we tested a single and relatively high dose of oxycodone in our analgesic test. In addition to our analgesic test being secondary measure beyond the scope of this study, we were also limited to using SFT from the typical nociceptive tests because, for instance, oxycodone causes a straub mouse tail rendering commonly used tail-flick test very challenging. However, it would be important to expand on this by establishing a dose-response curve, utilizing different feasible analgesic tests such as hotplate, and using a more traditional tolerance treatment paradigm. Lastly, we used the common opioid analgesic oxycodone in the present study. As other opioids have different receptor and immunoregulatory profiles, it is not clear how ubiquitous would be our findings to other opioids (58).

In conclusion, reductions in microglia expression had selective effects across the stages of Oxy dependence induction that were reflective of tolerance to nociceptive responses. The main hypothesis that microglia depletion would affect acute opioid withdrawal was not supported. The selective elevation of KC/GRO in the somatosensory cortex following chronic Oxy treatment and exacerbated by PLX requires further investigation and may prove to be a novel mechanism for delaying analgesic tolerance.

## Data Availability

The original contributions presented in the study are included in the article/Supplementary Material, further inquiries can be directed to the corresponding author.
