# Supplementary figures and images for: Microglial knockdown does not affect acute withdrawal but delays analgesic tolerance from oxycodone in male and female C57BL/6J mice
Source: Adv Drug Alcohol Res. 2022 Dec 16;2:10848. doi: 10.3389/adar.2022.10848 (PMC10880796; doi:10.3389/adar.2022.10848)

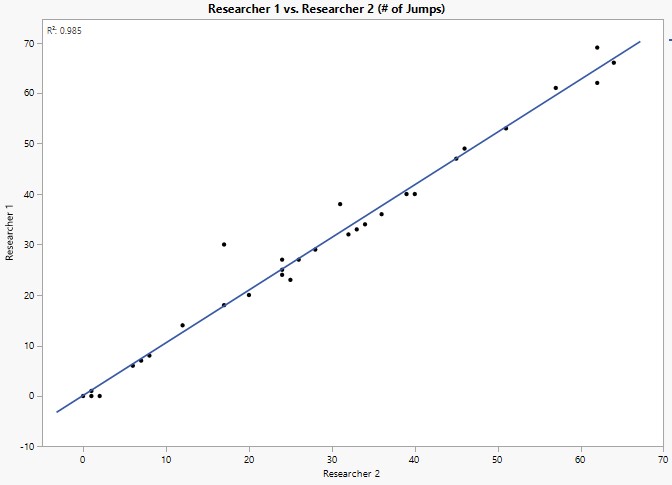

Supplement: Supplementary file 1 [file Image3.JPEG]

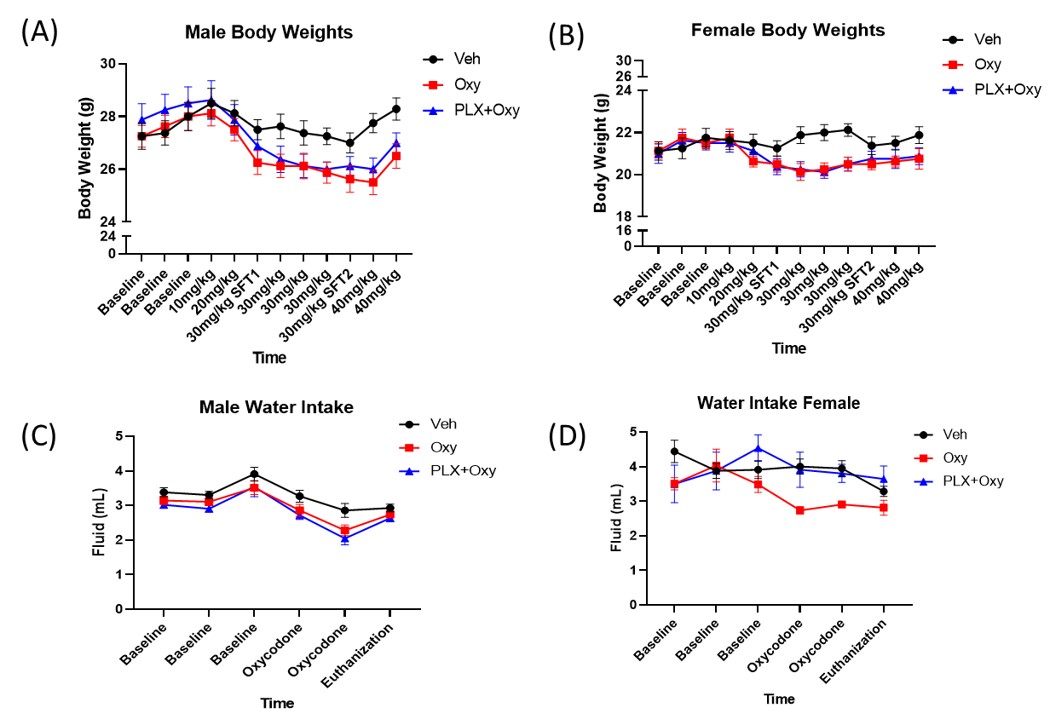

Supplement: Supplementary file 2 [file Image1.JPEG]

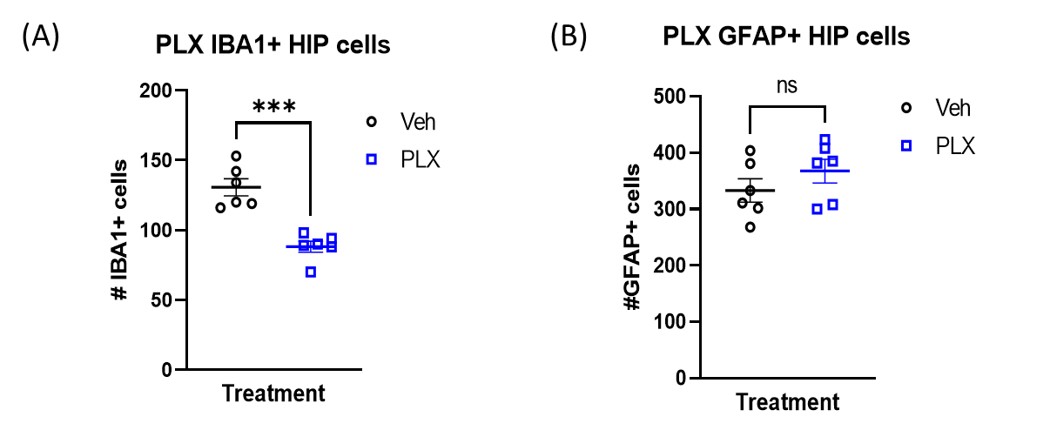

Supplement: Supplementary file 3 [file Image4.JPEG]

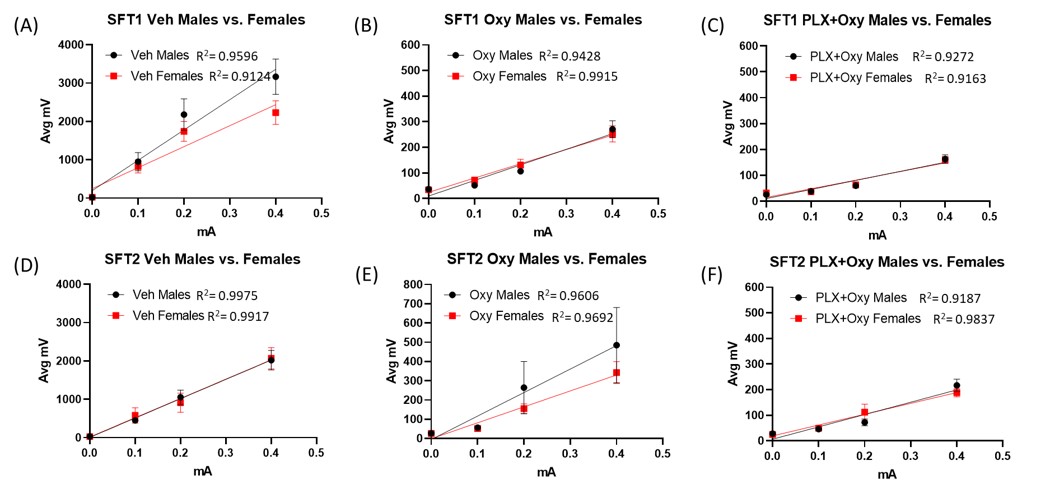

Supplement: Supplementary file 4 [file Image2.JPEG]

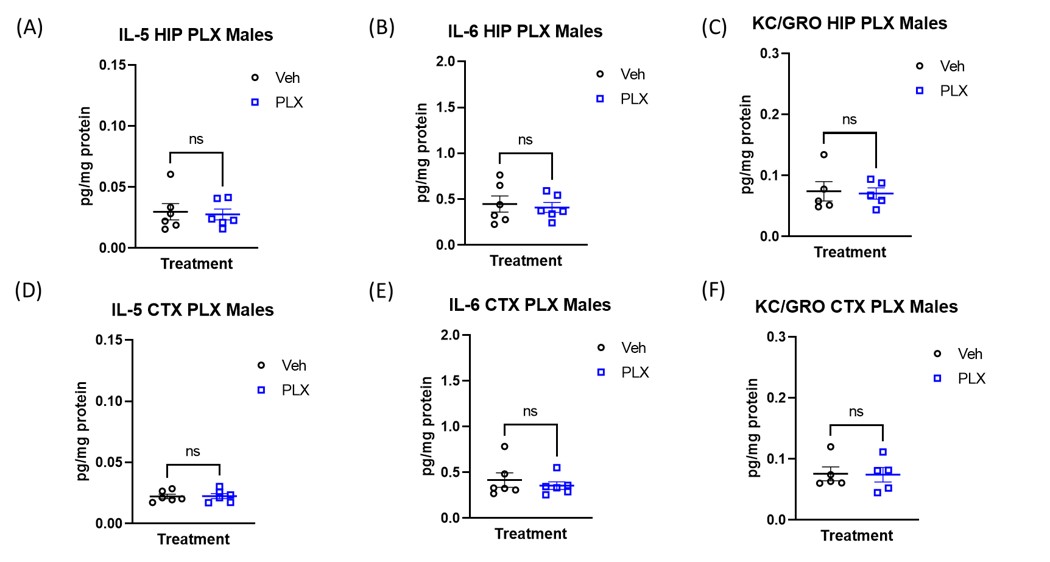

Supplement: Supplementary file 5 [file Image5.JPEG]

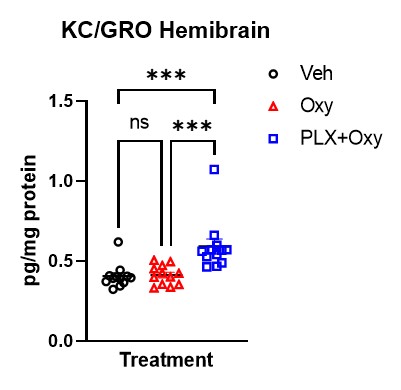

Supplement: Supplementary file 7 [file Image6.JPEG]
