# Supplementary material for: Microglial knockdown does not affect acute withdrawal but delays analgesic tolerance from oxycodone in male and female C57BL/6J mice
Source: Adv Drug Alcohol Res. 2022 Dec 16;2:10848. doi: 10.3389/adar.2022.10848 (PMC10880796; doi:10.3389/adar.2022.10848)
Supplement: Supplementary file 6 [file DataSheet1.docx]

**Supplementary Materials**

**Supplementary Figure 1.** Male and female body weight and water intake from Day 0 to Day 22. **(S1A)** Male body weight (grams) and **(S1B)** female body weights (grams) at 3 days prior to any Oxy (represent the three first baseline points in the graph) and every day during the Oxy treatment (Day 14 to Day 22). **(S1C)** Male water intake and **(S1D)** female water intake of 3 measurements prior to any Oxy (represent the first three baseline points in the graph) and every third day during Oxy treatment (Day 14 to Day 22) (n=7-8/group).

**Supplementary Figure 2.** SFT1/SFT2 male/female response comparison in response to Oxy. Male (M) and female (F) responses were compared on Day 16 (SFT1) or Day 20 (SFT2) at 30 mins after a 30 mg/kg Oxy injection. For the first shock flinch test (SFT1), we compared the male vs. female Veh groups (**S2A**; Linear regression slope: F (1, 60)=3.19, p=0.079; Intercept: F (1, 61)=3.33, p=0.073), Oxy groups (**S2B**; Linear regression slope: F (1, 60)=0.27, p=0.599; Intercept: F (1, 61)=0.16, p=0.688), and PLX+Oxy groups (**S2C**; Linear regression slope: F (1, 60)=0.10, p=0.748; Intercept: F (1, 61)=0.03, p=0.856) and found no significance between the sexes in each treatment. Linear regression equation: SFT1: Males vs. Females: Veh (**S2A**; M:y=7916*x+195.5; F:y=5480*x+247.5), Oxy (**S2B**; M:y=608.8*x+9.950; F:y=558.6*x+24.38), or PLX+Oxy (**S2C**; M:y=350.3*x+10.46; F:y=333.9*x+14.69). Similarly, we evaluated significance in the second shock flinch test (SFT2) between male vs. female for Veh groups (**S2D**; Linear regression slope: F (1, 56)=0.0002, p=0.988; Intercept: F (1, 57)=0.004, p=0.946), Oxy groups (**S2E**; Linear regression slope: F (1, 60)=0.93, p=0.337; Intercept: F (1, 61)=1.15, p=0.288), and PLX+Oxy groups (**S2F**; Linear regression slope: F (1, 60)=0.53, p=0.466; Intercept: F (1, 61)=0.05, p=0.814) and found no difference between the sexes. SFT2: Veh (**S2D**; M:y=5045*x+7.087; F:y=5032*x+18.00), Oxy (**S2E**; M:y=1221*x-5.513; F:y=827.7*x-1.150), or PLX+Oxy (**S2F**; M:y=481.7*x+6.575; F:y=423.3*x+19.56) (n=8/group for each graph). As a result, we collapsed across sexes in each of the SFT tests, this data is presented in Figure 3.

**Supplementary Figure 3.** Inter-rater correlation graph for jumping during withdrawal. Number of jumps from mice after a naloxone challenge one hour after receiving a 40 mg/kg Oxy or Veh injection on Day 21. Number of jumps were counted by 2 researchers blinded to treatment and the inter-rater correlation graphed for the number of jumps for each mouse regardless of treatment or sex. R^2^=0.985 (n=40 mice).

**Supplementary Figure 4.** Glial reactivity markers measured in male mice receiving PLX peanut butter balls or regular peanut butter balls. Hippocampus tissue was collected at Day 22 in a satellite animal group that received either PLX (no Oxy) or Veh (no Oxy) from Day0 to Day 22 and euthanized at approximately the same time of day as in the experiment where mice received both PLX and Oxy as presented in Figure 1. **(S4A)** Male IBA1+ cell counts in the hippocampus in PLX alone treated animals show a ~33% reduction in microglial staining. (Two-tailed t-test: t=5.878, df=10, p=0.0002) (n=6/group) **(S4B)** Male GFAP+ cell counts in the hippocampus shows the lack of change in astrocytic staining (Two-tailed t-test: t=1.164, df=10, p=0.271) (n=6/group).

**Supplementary Figure 5.** Cytokine measurements in male mice receiving PLX peanut butter balls or regular peanut butter balls. Brain hippocampus and cortical tissue were collected at Day 22 in a satellite animal group that received either PLX (no Oxy) or Veh (no Oxy) from Day 0 to Day 22 and euthanized at approximately the same time of day as in the experiment where mice received both PLX and Oxy as presented in Figure 1. PLX3397 did not affect cytokine expression in the hippocampus (HIP) or cortex (CTX). **(A)** IL-5 in HIP (Two-tailed t-test: t=0.2722, df=10, p=0.7910) (n=6/group). **(B)** IL-6 in HIP (Two-tailed t-test: t=0.3607, df=10, p=0.7258)(n=6/group). **(C)** KC/GRO in HIP (Two-tailed t-test: t=0.1987, df =8, p=0.8475)(n=5/group). **(D)** IL-5 in CTX (Two-tailed t-test: t=0.09136, df=10, p=0.9290)(n=6/group). **(E)** IL-6 in CTX (Two-tailed t-test: t=0.6821, df=10, p=0.5107)(n=6/group). **(F)** KC/GRO in CTX (Two-tailed t-test, t=0.09221, df =8, p=0.9288) (n=5/group).

**Supplementary Figure 6.** Quantitation of KC/GRO from mouse hemibrains in Vehicle, Oxycodone, or PLX+Oxycodone treatment male and female mice. Male and female mice C57BL/6J (n=12/group) were maintained on ad libitum PLX3397 (400pm; Envigo Teklad TD.210097; PLX76A) feed or regular feed (Envigo Teklad TD94096; AIN-76A) for 21 days. On day 14 of PLX3397 treatment, mice began their oxycodone treatment with an escalating dose of 10 mg/kg to 40 mg/kg or saline equivalent throughout the 8 days prior to euthanization. The escalating dose consisted of Day 14 (10 mg/kg), Day 15 (20 mg/kg), Day 16-20 (30 mg/kg) s.c. daily, twice a day at 0900 and 1700 hours. On Day 21 mice received a single (40mg/kg) s.c. at 0900 and allowed to go through spontaneous withdrawal for 24 hrs prior to perfusion and brain tissue collection. The left hemisphere was collected whole and homogenized with the same procedure, analyzed with the same proinflammatory kit, and normalized with BCA. Hemibrain cytokine analysis reveal a significant difference in the PLX+Oxy group compared to Oxy or Veh but Oxy alone compared to Veh did not differ (Two-way ANOVA: Interaction, F (2, 30)=2.03, p=0.148; Sex, F (1, 30)=0.019, p=0.8902; Treatment, F (2, 30)=11.61, p=0.0002; Veh vs. PLX+Oxy, p=0.0005; Oxy vs. PLX+Oxy, p=0.0008; Veh vs. Oxy, p=0.9851).
